# Dietary patterns of adults living in Ouagadougou and their association with overweight

**DOI:** 10.1186/1475-2891-9-13

**Published:** 2010-03-22

**Authors:** Elodie Becquey, Mathilde Savy, Peggy Danel, Hubert B Dabiré, Sylvestre Tapsoba, Yves Martin-Prével

**Affiliations:** 1 UMR 204-Nutripass, Institut de Recherche pour le Développement (IRD), BP 64501, F-34394 Montpellier cedex 5, France; 2 Doctoral School 0393 'Public Health: Epidemiology and Biomedical Information Sciences' Université Pierre et Marie Curie, Paris, France; 3 Institut Supérieur des Sciences de la Population, Ouagadougou, Burkina Faso; 4 Direction Nationale de la Nutrition, Ouagadougou, Burkina Faso

## Abstract

**Background:**

Urbanization in developing countries comes along with changes in food habits and living conditions and with an increase in overweight and associated health risks. The objective of the study was to describe dietary patterns of adults in Ouagadougou and to study their relationship with anthropometric status of the subjects.

**Methods:**

A qualitative food frequency questionnaire was administered to 1,072 adults living in two contrasted districts of Ouagadougou. Dietary patterns were defined by principal component analysis and described by multivariate analysis. Logistic regression was used to study their association with overweight.

**Results:**

The diet was mainly made of cereals, vegetables and fats from vegetable sources. The two first components of the principal component analysis were interpreted respectively as a "snacking" score and as a "modern foods" score. Both scores were positively and independently associated with the economic level of households and with food expenditures (p ≤ 0.001 for both). The "snacking" score was higher for younger people (p = 0.004), for people having a formal occupation (p = 0.006), for those never married (p = 0.005), whereas the "modern foods" score was associated with ethnic group (p = 0.032) and district of residence (p < 0.001). Thirty-six percent of women and 14.5% of men were overweight (Body Mass Index > 25 kg/m^2^). A higher "modern foods" score was associated with a higher prevalence of overweight when confounding factors were accounted for (OR = 1.19 [95% CI 1.03-1.36]) but there was no relationship between overweight and the "snacking" score.

**Conclusions:**

Modernisation of types of foods consumed was associated with the living conditions and the environment and with an increased risk of overweight. This should be accounted for to promote better nutrition and prevent non communicable diseases.

## Introduction

Urbanization is a fast-expanding phenomenon in developing countries. Projections are that urban populations in those countries will more than double between 2000 and 2025 [1]. Urbanization is not only synonymous of demographic changes but is also often regarded as a factor of economic and social growth. In particular, urban dwellers have better access to health services, water and sanitation facilities and education, though there are large differences between rich and poor, on the whole [2]. As far as food access is concerned, urban citizens experience less seasonal variations and have a wider range and a better availability of foods than their rural counterparts [2,3]. In low-income countries, urbanization is also often associated with changes in food habits, westernization of dietary patterns, lower levels of physical activity and overall changes in lifestyle [3]. Eating between main meals, buying street foods or processed foods and eating outside the home are frequent practices of urban citizens; also, consumption of meat, fat, salt and sweetened products increases [2-6]. This phenomenon, known as dietary transition, is accompanied by increased rates of overweight and obesity and growing risks of chronic diseases [7,8]. WHO estimated that 79% of the deaths due to non-communicable diseases occurred in developing countries in the early 2000's [9]. This put a strain on health systems which have to cope with these diseases while they still have to address infectious diseases and undernutrition. All developing countries experience dietary and nutritional transitions, at least in urban areas, but at a more or less advanced stage [10,11]. However the association between dietary changes and higher rates of overweight is not fully explained. Changes in food habits, dietary patterns, type of foods eaten and an increase in energy intake are often mentioned but there is no consensus on their respective contribution to overweight [3,12].

In Burkina Faso (West Africa), urban population has been multiplied by seven since 1975 and urban annual growth rate is very high: + 5.15% per year over the 2000-2005 period [13,14]. In 2003, results of a Demographic and Health Survey showed that 33.1% of women living in Ouagadougou, the capital city of Burkina Faso, were overweight or obese, as compared to only 3.8% of their rural counterparts. In this city, however, there were still 12.4% of under-five children who were wasted and 16.1% who were stunted [15]. Food prices, food provisioning and transformation have been studied in Ouagadougou [3,6,16,17] but information is still needed to characterise food habits and dietary patterns and on how they relate to individuals' anthropometric status. Dietary patterns are useful to describe the whole diet, including the potential synergetic effects of foods or nutrients; such an analysis also takes advantage of the collinearity between nutrients or foods when studying the relationships of diet with health outcome [18,19]. As Burkina Faso is still at an early stage of the nutritional transition, information on dietary patterns and their anthropometric outcome is essential to help design intervention strategies aiming at preventing adverse consequences of urbanization.

The objectives of our study were: (i) to identify and describe dietary patterns according to individual characteristics; (ii) to examine their relationship with the anthropometric status of the subjects.

## Methods

### Sampling

A cross-sectional survey took place in April/May 2005 (dry season) in two districts of Ouagadougou, on a sub-sample of the population monitored by the Demographic Monitoring System carried out by the *Institut Supérieur des Sciences de la Population *(ISSP) [Higher Institute of Population Sciences]. These districts have been purposely chosen as different: Wemtenga is a structured district (i.e. a district with amenities, in particular with electricity and water supply) of about 2,500 inhabitants, located near the centre of the town; Taabtenga is a non-structured district (i.e. with no amenities, because of rapid spontaneous urbanization) of about 3,500 inhabitants, located at the periphery of the city. In each district, 300 women and 300 men aged 20 to 65 years were randomly selected from an exhaustive list of inhabitants. Both study areas and sampling technique have already been described elsewhere [20]. The difference in sampling fractions between the two districts was taken into account in all analyses through the attribution of corresponding weights.

### Dietary data collection

A qualitative food frequency questionnaire (q-FFQ) was carefully designed, pre-tested on a similar population and then administered to all subjects of the study. This q-FFQ included all locally available foods, identified by their local names: 27 dishes, 9 sauces, 30 snacks and 16 drinks. Interviewees were asked to spontaneously recall all dishes, sauces, snacks and drinks they had consumed over the previous week and to estimate their frequency of consumption. They were then prompted for each item that had not been cited. When items were not consumed during the previous week but were part of the usual diet, and for rarely consumed or seasonal foods, frequencies of consumption per month were estimated and were then turned into frequencies per week over a year. Any food item that was not initially included into the questionnaire but was consumed by a subject during the survey period was then added to the list. The composition of mixed dishes, sauces or snacks was detailed by the interviewees. Then all food items were coded into one or more food groups, using the following list of 22 food groups: *cereals, roots/tuber, nuts and seeds, beans and pulses, vitamin A rich fruits and vegetables, other fruits, other vegetables, fatty/processed meats, non fatty meats and poultry, liver, fresh fish, dried fish, eggs, milk/yoghurt, cheese, animal source fats, vegetal source fats, red palm oil, fried foods, sugar/sweetened products, sweetened drinks, alcohol*. Frequencies of consumption of each food group per week were finally computed and used in the analyses.

### Dietary data analysis

Dietary patterns were determined by principal component analysis (PCA) performed on normalized frequencies of consumption of the 22 food groups (z-scores). The PCA method allows data reduction by identifying, in a multidimensional space, components that summarize the maximum of variability between subjects [21,22]. These components can be interpreted according to initial and supplementary variables that are well correlated with them. The normalized frequencies of consumption of the 27 dishes, 9 sauces, 30 snacks and 16 drinks, as recalled by the q-FFQ, were introduced as supplementary variables (i.e. not taken into account for the computation of the components) to help with the interpretation of results. Only components exhibiting an eigenvalue >2 were considered. This was the case for the two first components. This choice, though somehow arbitrary, was further confirmed by the shape of the screeplot of variance associated with each component, and by their interpretability. As the two first components were easily interpretable, rotation was neither used nor tried [21,23]. Scores of individuals for the two first components of the PCA were used to characterize the two main patterns of food consumption that distinguished people's diets in our sample.

### Household and individual characteristics

A section of the questionnaire focused on social, economic and demographic characteristics of households and individuals, and another section focused on dietary habits such as the number of meals per day, snacking habits, eating outside the home.

At the household level, an economic score was constructed by multiple correspondence analysis [24] using the following variables:*number of persons per room, quality of the wall and roof materials, electricity, piped water, source of drinking water, type of toilets and shower, waste evacuation, owning of TV set, telephone, refrigerator, video tape recorder and means of transportation*. This method projects all subjects on to a multidimensional space according to modalities taken by the variables of interest. Modalities associated with a low economic status (no shower, no kitchen, poor quality roof and walls...) were projected negatively on the first factor and modalities associated with a good economic status (car, shower, many goods, good quality roof and walls etc) were projected positively. The score of every household on the first factor of the multiple correspondence analysis was interpreted as a continuous economic score which has been divided into tertiles to determine low, medium or high economic level.

An individual physical activity score was also computed, based on the frequency of various physical activities: *walking, having a physically-demanding profession, practicing a sport, carrying water, crushing cereals, hand-washing clothes, and other physically-demanding activities*. Each variable was arbitrarily given a score (0 if never; 1 if sometimes; 2 if often) and the final score was the sum of them (range 0-21). The score has been divided into tertiles to determine low, medium or high physical activity level.

Home food stocks were estimated by the subjects from the quantities of all food items stocked at home (bought at least two days before) and their price. All prices were added up with the help of the field workers to obtain a monetary value of home food stocks.

### Anthropometrics

Anthropometric measurement procedure met the WHO recommendations [25]. Measurements were performed at home by field workers. Height was measured to the nearest mm with locally-made devices equipped with height gauges (SECA 206 Bodymeter). Weight (to the nearest 100 g) and body fat percentage (BFP) were measured on scales with a maximum weighing capacity of 130 kg including a foot-to-foot impedance analyser (Bodymaster™, SEB Group, France). The BFP was corrected to take into account the specificity of body composition of African populations [26]. Overweight was defined by a Body Mass Index (BMI) superior to 25 kg/m^2^. Overfatness, or excess of body fat, was defined by a BFP superior to the gender and age-specific cut-offs defined by Gallagher for African-American subjects [27]. Pregnant women (4.6%) and individuals with a physical handicap (1.2%) were excluded from the analyses using anthropometrics.

### Data management and analysis

Epi-data software, version 3.1 (The Epi-Data Association, Odense, Demark), was used for data entry, which was made in double to limit data-entry errors. Further data quality checks, data cleaning and statistical analyses were performed with the SAS system, version 9.1 (SAS Institute Inc., Cary, NC, USA). Individuals with incomplete observations for the FFQ (n = 12) and one outlier were excluded from the analysis.

The description of the two dietary patterns according to food habits, food access and socio-demographic characteristics was performed by multivariate linear regression using a manual backward stepwise procedure. The adjusted mean and the standard error of the mean were calculated for each characteristic. Associations between overweight or overfatness and the two dietary patterns were studied by logistic regression, controlling for confounding factors. As the two scores were continuous variables, values of the odds ratios are associated with an increase of one point of the score. The confounding factors were defined as variables that were associated with both anthropometrics and dietary patterns with a type I error of 0.20 and that did not belong to the causal pathway between them. In all other analyses, the level of significance was set at p < 0.05.

### Ethics

The study protocol received the approval of the National Committee of Ethics (Ministry of Health) and written informed consent was obtained from all participants.

## Results

One thousand and seventy-two individuals aged 15-65 years were surveyed: 276 women and 261 men lived in Wemtenga (structured district) and 281 women and 254 men lived in Taabtenga (unstructured district).

**Figure 1:**
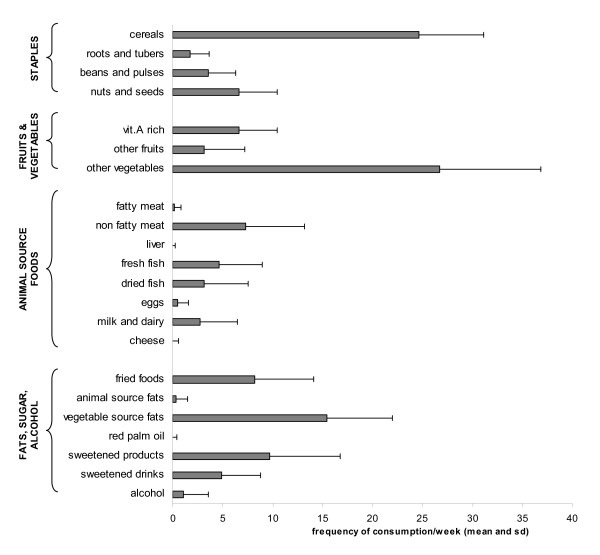
**Mean weekly frequency of consumption of the 22 food sub-groups**.

The diet was mainly made of cereals and vegetables (more than 3 times daily) and to a lesser extent of oil from vegetable sources (more than twice daily) (figure 1). Sweetened products, fried foods, non-fatty meats, nuts and seeds and vitamin A-rich fruits and vegetables were also rather frequently consumed, approximately once a day. Other fruits were consumed less frequently (3 times a week). Other animal-source foods were also less frequently consumed particularly dairy products (3 times a week) and eggs (less than once a week).

**Figure 2:**
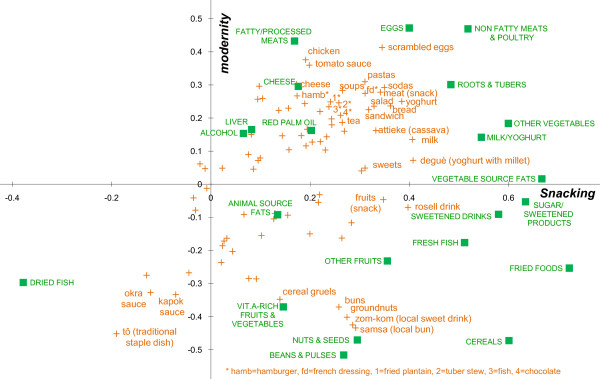
**Plot of the factor loadings of food groups (active variables) and food items (supplementary variables)**. Active and supplementary variables are plotted on the first two components of the PCA. All active variables (food groups) appear in green and capital letters. Supplementary variables (food items) appear in orange and small letters. For clarity, only well represented food items, i.e. items whose sum of cos^2 ^on the first two components was superior to the mean, are named in the graph. Other food items were not informative for interpretation and were not labelled.

The two first components of the PCA accounted for 28.5% of the total variance in the data set. The first component, which explained most of the variability between individual dietary consumptions (19% of the total variance), was interpreted as a pattern of snacking: first, almost all food groups and most of foods were positively represented on this first component which means that subjects with high positive scores on the first component tended to consume frequently many sorts of food groups/foods and thus had a high frequency of food intakes; second, food items with the highest scores on this first component were foods commonly consumed outside the main meals (déguè, milk, sweetened drinks, yoghurt, bread, fruits...) (table 1, figure 2. Alternatively to figure 2, see additional file 1, table S1 which presents factor loadings of the supplementary variables on the first two principal components). In this study, snacking will thus be defined as "frequent food consumption outside the main meals". The second component opposed traditional dishes and sauces (tô, okra and kapok sauces) and local snacks (groundnuts, local buns and drinks) to more modern types of foods or preparations (scrambled eggs, chicken, tomato sauce, pastas, cheese, meat, sodas, soup, French dressing, hamburger). This is why the second component was interpreted as a pattern of modernity of the *type *of foods consumed (regardless food habits).

**Table 1:** Factor loadings of the food groups on the first two principal components identified ^(a)^

Food groups	Snacking	Modernity
fried foods	**0.72**	-0.25
vegetable source fats	**0.67**	0.02
sugar/sweetened products	**0.63**	-0.05
Cereals	**0.60**	**-0.47**
other vegetables	**0.60**	0.18
sweetened drinks	**0.58**	-0.09
milk/yoghurt	**0.54**	0.14
non fatty meats and poultry	**0.52**	**0.47**
fresh fish	**0.51**	-0.18
roots and tubers	**0.48**	0.30
Eggs	0.40	**0.47**
other fruits	0.36	-0.23
nuts and seeds	0.30	**-0.47**
beans and pulses	0.27	**-0.52**
red palm oil	0.20	0.16
Cheese	0.18	0.30
fatty/processed meats	0.17	**0.43**
vitamin A-rich fruits and vegetables	0.15	**-0.37**
animal source fats	0.13	-0.09
Liver	0.08	0.16
Alcohol	0.07	0.15
dried fish	-0.38	-0.30

^(a) ^loadings for variables whose contribution to the variance of the component is superior to the mean are shown in bold

To quantify the two dietary patterns, the loading of individuals on the first component of the PCA was used as a "snacking" score (minimum = -4.74; maximum = 10.12), and the loading on the second component was used as a "modern foods" score (minimum = -4.14; maximum = 8.11).

Regarding food habits and access, subjects declaring the highest occurrences of food intakes between meals and the highest number of meals eaten per day had a significantly higher "snacking" score (table 2). This score was significantly higher when the monetary value of food stocks, the daily household food expenditure per person or the economic score were higher (p ≤ 0.001 for each variable). Subjects working in the formal sector, younger subjects, single subjects, and Muslims had significantly higher "snacking" scores. District of residence, level of education, ethnic group and consumption of dishes outside the home were not associated with the "snacking" score after adjustment for confounding variables.

**Table 2:** Characteristics associated with the "snacking" score and with the "modern foods" score ^(a)(b)^

		%	Adjusted mean ± SEM^(c) ^of the "snacking" score	*p*	Adjusted mean ± SEM^(c) ^of the "modern foods" score	*p*
*usual number of meals eaten per day*						
	one or two	44	-0.40 ± 0.09	<0.001	0.36 ± 0.06	<0.001
	three or more	56	0.24 ± 0.08		-0.33 ± 0.05	
*consumption of foods between meals*						
	<2 times a week	29	-0.82 ± 0.11	<0.001	0.24 ± 0.07	<0.001
	2 to 6 times a week	44	0.02 ± 0.09		-0.17 ± 0.05	
	every day	26	0.79 ± 0.12		-0.12 ± 0.07	
*consumption of dishes outside the home*						
	never	22	-0.13 ± 0.13	0.068		
	once a week or less	20	-0.32 ± 0.13			
	2 to 6 times a week	15	0.09 ± 0.15			
	every day	44	0.10 ± 0.09			
estimation of the monetary value of food stocks*						
	no stocks	36	-0.31 ± 0.10	<0.001	-0.06 ± 0.06	0.059
	]0 - 10,000] cfa	13	-0.10 ± 0.16		0.18 ± 0.10	
	]10.000 - 20,000] cfa	25	-0.16 ± 0.11		-0.16 ± 0.07	
	]20.000 - 30,000] cfa	17	0.26 ± 0.14		-0.02 ± 0.09	
	more than 30,000 cfa	9	0.87 ± 0.19		0.11 ± 0.12	
daily household food expenditure per person*						
	[0 - 100[cfa	31	-0.33 ± 0.10	<0.001	-0.24 ± 0.07	<0.001
	[100 - 200[cfa	39	-0.10 ± 0.09		-0.05 ± 0.06	
	[200 - 500[cfa	23	0.23 ± 0.12		0.15 ± 0.08	
	500 cfa and more	7	0.81 ± 0.23		0.36 ± 0.14	
*economic score*						
	low	39	-0.29 ± 0.10	0.001	-0.28 ± 0.08	0.001
	medium	34	-0.07 ± 0.10		-0.02 ± 0.06	
	high	27	0.37 ± 0.12		0.30 ± 0.11	
*district*						
	non structured	58			-0.25 ± 0.06	<0.001
	structured	42			0.30 ± 0.09	
*occupation*						
	none	36	-0.15 ± 0.10	0.006		
	informal sector ^(d)^	49	-0.10 ± 0.08			
	formal sector	15	0.43 ± 0.16			
*ever attended school*						
	no	43			-0.12 ± 0.06	0.090
	yes	57			0.03 ± 0.05	
*Age*						
	< 25 y	24	0.26 ± 0.13	0.004		
	25-29 y	25	0.03 ± 0.12			
	30-39 y	28	0.01 ± 0.11			
	40 y and more	23	-0.47 ± 0.13			
*marital status*						
	single	37	0.28 ± 0.11	0.005	0.08 ± 0.07	0.092
	couple	60	-0.22 ± 0.08		-0.10 ± 0.05	
	widowed/divorced	3	-0.29 ± 0.34		-0.12 ± 0.21	
*religion*						
	muslims	61	0.11 ± 0.07	0.007	-0.16 ± 0.05	<0.001
	catholics	33	-0.27 ± 0.10		0.17 ± 0.06	
	Others	6	-0.26 ± 0.24		0.12 ± 0.15	
*ethnic group*						
	mossi	67			-0.09 ± 0.04	0.032
	other than mossi	33			0.08 ± 0.06	

^(a) ^results of multivariate analyses. Variables with p > 0.10 were not included in the model and not reported in the table. thus appear as blank cells

^(b) ^characteristics tested but not significantly associated with the one or the other score: gender, rural/urban place of birth, supplementary income

^(c) ^SEM: standard error of the mean

^(d) ^occupations within the informal sector can be various, but their common point is that they are not declared and thus neither taxed nor monitored by the government. This often corresponds to less stable occupation than within the formal sector

* equivalent in local currency (cfa Franc). 1 Euro = 656 cfa Francs

The "modern foods" score was significantly higher when the number of meals eaten per day was lower and when frequency of food intakes between meals was less frequent (p < 0.001). It was positively associated with the daily household food expense per person (p < 0.001) and the economic score (p = 0.001). Subjects who lived in the structured district, who did not belong to the Islam religion nor to the Mossi ethnic group had a higher "modern foods" score (p < 0.001, p < 0.001 and p = 0.032 respectively). Consumption of dishes outside the home, food stocks, occupation, schooling, age and marital status were not associated with the modern aspect of foods consumed after adjustment for confounding variables.

In our sample, the mean BMI was 24.2 ± 5.0 kg/m^2 ^among women and 21.8 ± 3.1 kg/m^2 ^among men; 36.0% of women and 14.5% of men were overweight or obese. The mean BFP was 36.1 ± 8.6% among women and 16.3 ± 5.3% among men; 56.1% of women and 17.3% of men were overfat. The "snacking" score was neither associated with overweight nor with overfatness, after adjustment for confounding factors (table 3). On the other hand, after adjustment for potential confounding factors, an increase of one unit in the "modern foods" score was associated with an increased risk of being overweight (OR = 1.19 [95% CI 1.03-1.36], p = 0.018) or overfat (OR = 1.14 [95% CI 1.00-1.29], p = 0.044). No significant interaction was found between gender and the "modern foods" score or the "snacking" score, neither for the prediction of overweight nor for the prediction of overfatness.

**Table 3:** association between the "snacking" score and the "modern foods" score and overweight and overfatness ^(a)^

		N	"snacking" score	"modern foods" score
OR^(b) ^for overweight	unadjusted	1060	1.01 [0.94-1.09] (p = 0.703)	1.19 [1.08-1.32] (p <0.001)
	adjusted^(c)^	867	1.04 [0.95-1.13] (p = 0.424)	1.19 [1.03-1.36] (p = 0.018)

OR^(b) ^for overfatness	unadjusted	1060	1.03 [0.96-1.10] (p = 0.340)	1.11 [1.02-1.22] (p = 0.015)
	adjusted^(c)^	911	1.03 [0.95-1.12] (p = 0.483)	1.14 [1.00-1.29] (p = 0.044)

^(a) ^multiple logistic regression model

^(b) ^OR: Odds Ratio associated with an increase of one point of the score

^(c) ^adjusted for the following variables: age, gender, physical activity, ethnicity, rural/urban place of birth, marital status, economic level, professional occupation, schooling and district. The interaction score*gender was not included in the final model because it was not significant

## Discussion

In this urban African setting, subjects' diet was based on cereals and vegetables as it is traditionally the case in Burkina Faso. Our data showed that many food groups were rarely eaten, particularly foods from animal sources such as dairy products and eggs. The variety of the diet was rather low, though higher than that of people living in rural Burkina Faso [20]. While the general diet remained very traditional, the principal component analysis revealed that the two dietary patterns responsible for most variability between individual's diets could be interpreted as a "snacking" score (first component of the PCA) and a "modern foods" score (second component of the PCA). These two patterns of food consumption are typically urban. The "modern foods" score was associated to a higher risk of being overweight and overfat whereas the "snacking" score was not.

We have to acknowledge some limitations of our study. First, the sample was not representative of the whole population of Ouagadougou. However, we compared socio-demographic and economic data of the two districts already available from the Demographic and Health Monitoring System (DHMS) to the same data from the representative sample of Ouagadougou used by the most recent DHS (2003) [28]. When selecting an equal number of subjects from each district, the mean values obtained matched correctly, suggesting that our sample covered a large social and demographic diversity of individuals living in the city. Hence, we assume that our sample also caught a maximum of variety in diets and food patterns that should not be dramatically different from those observed in the whole population of Ouagadougou. Second, our q-FFQ was pre-tested in a population similar to our study sample but was not validated against another dietary method or biomarkers. However, the purpose of this study was to explore food consumption in Ouagadougou in order to draw a very first picture of diets to fill a gap of knowledge in such a setting. The pre-test revealed that our q-FFQ was acceptable and understandable by the subjects. In addition, the food grouping used had been proved to be associated with several socio-economic and demographic characteristics of women of the same sample [20]. It is also often argued that the lack of quantification of food intake is a major limit of the FFQ method. Nevertheless, FFQ has been identified a quick and valid tool to assess dietary consumption that, when combined with factor analysis, allows studying dietary patterns in relation to health outcomes, even without adjustment on energy intake or portion size [18,29]. Given the challenge of estimating portion sizes in such a setting and given the explorative purpose of the study, development of a semi-quantitative version of our questionnaire was not considered. In addition, our q-FFQ was designed to minimise some other sources of bias by means such as the accuracy of the list of foods, the aggregation of frequencies from single items or the frequency options which could be expressed weekly or monthly [30]. The semi-open interview method allowed staying close to what subjects really ate and allowed including all sort of foods even if very specific. Finally, in order to account for the two major co-factors of dietary intake, namely households' economic level and individuals' physical activity, we relied on proxy scores that were not validated as such. However, the economic score was constructed using a standard method recommended by several authors [31-33]. As far as the physical activity score is concerned, we ensured that our proxy was significantly and independently associated with overweight and overfatness, in the expected direction, in the models studying the relationships of these variables with both the "snacking" and the "modern foods" scores (results not shown).

*A-posteriori *methods to derive dietary patterns have some limitations: first, researchers have to take quite subjective decisions at various stages of the process; second, results are very population-specific and not reproducible across different sample [21,34]. However, these methods are very useful to summarize behaviours from many variables in a population where no previous knowledge exists; hence the choice of PCA to perform dietary pattern analysis in our sample, which was driven by the fact that very few data are available about food consumption patterns in Ouagadougou. It was therefore difficult to apply an *a-priori *method such as the use of pre-defined indices. Indeed, these types of indices are based on existing knowledge about relationship between a type of diet and health outcomes and must also be adapted to the specific population behaviours [19]. To our knowledge, such an index has never been validated in a Sahelian urban area. Among *a-posteriori *methods, we chose PCA over cluster analysis to insist on similarities rather than on differences between diets. However, both methods have proved to reveal similar dietary patterns [35].

Our results showed interesting relationships between the living conditions of individuals and the level of the two scores characterizing their diet. First, snack consumption was associated with the less structured living conditions such as those of younger and single subjects: older and married subjects are more likely to have an organised life with planned meals. Likewise, working people, especially those working in the formal sector, ate snack foods more frequently, probably due to the little time available and the organisation of the day outside their home. Second, while the district of residence was not associated with the "snacking" score, it was associated with the "modern foods" score. These results mean that snacking was encountered in both districts but *types *of snack were different: traditional snacks in the non structured district and more modern snacks in the structured one. This highlights the role of environment and food availability in the choice of foods consumed, as described by many authors [36,37]. Likewise, ethnicity and religion had an impact on the modernity of foods consumed: subjects from the native ethnic group (Mossi) had more traditional diets than the non native, as well as Muslims as compared to other religions. Finally, the economic level was positively associated with both scores, suggesting that the poorest people can only afford traditional and staple diets. This interpretation is reinforced by the positive relationship between the two scores and the household food expenditure: whatever the household's economic level, snacking or eating modern foods is more costly.

Although definitions and interpretation of traditionalism, modernity or snacking vary quite a lot across dietary pattern studies, other studies described a pattern close to our description of the traditional/modern foods pattern, while very few studies identified a snacking pattern. In a review summarizing 30 studies that reported dietary patterns derived from factor analysis [29], 19 of them identified either a traditional or a western pattern, and sometimes both. Healthier patterns (i.e. not the western pattern) were associated with higher income, education and age, but we have to acknowledge that most of these studies were performed in developed countries. Associations between dietary patterns and living conditions or socio-demographic characteristics appear to be context-dependent. In the same review, only one out of the 30 studies identified a snacking pattern, but it was amongst young children living in a geographically and culturally different area compared to our study area [38]. Recent studies describing diets derived from data-driven methodologies and performed in West Africa are scarce. No other study using PCA to derive dietary patterns could be found in similar populations. The closest study we identified was conducted in urban Benin and authors derived diets from cluster analysis [39]. Its results were in line with ours: the traditional diet in Cotonou was characterised by high intakes of cereals and the transitional diet by high intakes of bread and pastas, animal source foods, milk products, fats and sweets. This is quite similar to the description of the "modern foods" score we identified in Ouagadougou. Also, a positive relationship between socio-economic score and modernity of food intake was identified in Cotonou, as in Ouagadougou.

One other important result of our study is that the "modern foods" score was positively associated with overweight and with overfatness. Similar results were obtained with BMI and BFP used as continuous variables (results not shown). This is in line with the concept of dietary transition described by the literature, and suggests that in Ouagadougou, changes in types of food eaten would be one explanation among others for the nutrition transition. Many other studies, in different settings, focused on the relationship between dietary patterns and anthropometric status. A review reported that 19 studies out of 30 showed a relationship between western food patterns and overweight [12]. In urban Brazil, another study demonstrated that a "traditional" diet, constructed by factor analysis, was associated with a lower risk of overweight and obesity [40].

In Ouagadougou, more in-depth investigation is still required to identify what exactly in the consumption of modern types of foods leads to increased overweight. Possible explanations are quantities consumed, food habits, macronutrient balance or energetic content of modern types of foods. To investigate all these possible explanations, a quantitative study describing the nutrient content of modern foods is required. The assessment of their energy and micronutrient densities would help to clarify the relationship between the "modern foods" pattern and overweight. Since the "modern foods" score was negatively associated with the number of main meals in the day and with the frequency of food consumption in between, eating more modern types of foods does not seem to be explained by a multiplication of food intake occasions. This is in line with the fact that the "snacking" score, which is independent of the "modern foods" score by construction, was not associated with an increased risk of overweight. Alternate hypothesis would be that quantities consumed at each food intake are more substantial and/or that most of modern foods are energy-dense, leading to unbalanced diet. The latter hypothesis is supported by a study conducted in Ouagadougou, which showed that obesity was associated with a higher consumption of micronutrient-, protein-, and sugar-rich foods [41]. An interesting hypothesis was also suggested from the study in Cotonou [39]. The authors explained that the transitional diet was not a drastic replacement of traditional foods by western ones, but more the result of adding western foods to a traditional diet. This scenario could be transposed to Ouagadougou and could therefore explain part of the phenomenon.

## Conclusions

Our study showed that in a setting where dietary patterns remain largely traditional, there was some evidence of a higher risk of being overweight and overfat associated with consumption of modern types of foods but not with snacking. While consumption of modern foods, known to be more frequent in urban settings, appeared to be still limited in our sample, it must be noticed that another study in Ouagadougou identified worrying rates of non communicable food related diseases [41,42]. This highlights the relevance of studying food patterns in such a context. Such studies may help implementing appropriate nutritional policies and programs to address the double burden of malnutrition, especially in West-African countries which are still at an early stage of their nutritional transition.

## Competing interests

The authors declare that they have no competing interests.

## Authors' contributions

Y.M-P., M.S., S.T. and H.D. designed the study. M.S., P.D. and Y.M-P. collected the data. E.B. and Y.M-P. performed the data analysis and drafted the manuscript. All authors participated in the interpretation and discussion of the results and critically revised the manuscript. All authors read and approved the final manuscript.

## Supplementary Material

Additional file 1 **Factor loadings of the food items (supplementary variables) on the first two principal components identified**. Table alternative to figure 2 presenting the factor loadings of the food items on the first two principal components. Click here for file
