# Supplementary material for: Dietary patterns of adults living in Ouagadougou and their association with overweight
Source: Nutr J. 2010 Mar 22;9:13. doi: 10.1186/1475-2891-9-13 (PMC2848625; doi:10.1186/1475-2891-9-13)
Supplement: Additional file 1 — Factor loadings of the food items (supplementary variables) on the first two principal components identified. Table alternative to figure 2 presenting the factor loadings of the food items on the first two principal components. [file 1475-2891-9-13-S1.PDF]

**Additional file 1, table S1: Factor loadings of the food items (supplementary variables) on the first two principal components identified [(a)]**

| Food items | Snacking | modernity |
|---|---|---|
| scrambled eggs | **0.34** | **0.41** |
| samsa (local bun) | **0.29** | **-0.43** |
| zom-kom (local sweet drink) | **0.29** | **-0.43** |
| tô (traditional staple dish) | -0.19 | **-0.45** |
| groundnuts | **0.27** | **-0.40** |
| yoghurt | **0.38** | **0.25** |
| sodas | **0.35** | **0.29** |
| buns | **0.26** | **-0.37** |
| meat (as snack) | **0.34** | **0.28** |
| pastas | **0.31** | **0.31** |
| bread | **0.36** | **0.24** |
| milk | **0.41** | 0.13 |
| chicken | 0.19 | **0.38** |
| french dressing | **0.31** | **0.27** |
| deguè (yoghurt with millet) | **0.41** | 0.07 |
| tomato sauce | 0.20 | **0.36** |
| salad | **0.33** | **0.24** |
| rosell drink | **0.40** | -0.07 |
| sandwich | **0.32** | **0.23** |
| meat or fish soups | **0.27** | **0.28** |
| cereal gruels | 0.14 | **-0.35** |
| attieke (cassava semolina) | **0.33** | 0.16 |
| tuber stew | **0.26** | **0.25** |
| fruits (as snack) | **0.35** | -0.05 |
| okra sauce | -0.12 | **-0.33** |
| cheese | 0.17 | **0.30** |
| fried plantain | **0.24** | **0.25** |
| kapok sauce | -0.07 | **-0.33** |
| chocolate | **0.26** | 0.21 |
| fish | **0.24** | **0.23** |
| tea | **0.26** | 0.19 |
| hamburger | 0.17 | **0.27** |
| sweets | **0.31** | 0.05 |
| prawn chips | **0.24** | 0.20 |
| biscuits | **0.27** | 0.16 |

[(a)] for clarity, only well represented food items, i.e. items whose sum of $\cos^2$ on the first two components was superior to the mean, are named in the table. Other food items were not informative for interpretation. Loadings for variables whose contribution to the variance of the component is superior to the mean are shown in bold.
